# Prognostic impact of *KRAS*, *NRAS*, *BRAF*, and *PIK3CA* mutations in primary colorectal carcinomas: a population-based study

**DOI:** 10.1186/s12967-016-1053-z

**Published:** 2016-10-13

**Authors:** Grazia Palomba, Valentina Doneddu, Antonio Cossu, Panagiotis Paliogiannis, Antonella Manca, Milena Casula, Maria Colombino, Annamaria Lanzillo, Efisio Defraia, Antonio Pazzola, Giovanni Sanna, Carlo Putzu, Salvatore Ortu, Mario Scartozzi, Maria Teresa Ionta, Giovanni Baldino, Giuseppina Sarobba, Francesca Capelli, Tito Sedda, Luciano Virdis, Michela Barca, Giulia Gramignano, Mario Budroni, Francesco Tanda, Giuseppe Palmieri

**Affiliations:** 1 Institute of Biomolecular Chemistry, CNR, Sassari, Italy; 2 Department of Surgical, Microsurgical and Medical Sciences, University of Sassari, Viale San Pietro 43, 07100 Sassari, PC Italy; 3 Oncology Unit, Businco Hospital, Cagliari, Italy; 4 Medical Oncology Unit, University—Hospital of Sassari (AOU), Sassari, Italy; 5 Oncology Unit, Local Health Agency, Olbia, Italy; 6 Department of Medical Oncology, University of Cagliari, Cagliari, Italy; 7 Oncology Unit, Civil Hospital, Alghero, Italy; 8 Oncology Unit, Zonchello Hospital, Nuoro, Italy; 9 Oncology Unit, Local Health Agency, Oristano, Italy; 10 Oncology Unit, Local Health Agency, Carbonia-Iglesias, Italy; 11 Oncology Unit, Local Health Agency, Lanusei, Italy

**Keywords:** Colorectal cancer, *KRAS*, *NRAS*, *BRAF*, *PIC3CA*

## Abstract

**Background:**

Activation of oncogenes downstream the EGFR gene contributes to colorectal tumorigenesis and determines the sensitivity to anti-EGFR treatments. The aim of this study was to evaluate the prognostic value of *KRAS, BRAF, NRAS* and *PIK3CA* mutations in a large collection of CRC patients from genetically-homogeneous Sardinian population.

**Methods:**

A total of 1284 Sardinian patients with histologically-proven diagnosis of colorectal carcinoma (CRC) and presenting with metastatic disease were included into the study. Genomic DNA was isolated from formalin-fixed, paraffin-embedded primary tumour tissue samples of CRC patients and screened for mutations in *RAS* and *BRAF* genes, using pyrosequencing assays, and in *PIK3CA* gene, using automated DNA sequencing assays.

**Results:**

Overall, mutation rates were 35.6 % for *KRAS*, 4.1 % for *NRAS*, and 2.1 % for *BRAF*. Among available DNA samples, 114/796 (14.3 %) primary CRCs were found to carry a mutation in the *PIK3CA* gene. In this subset of patients analysed in all four genes, a pathogenetic mutation of at least one gene was discovered in about half (378/796; 47.5 %) of CRC cases. A mutated *BRAF* gene was found to steadily act as a negative prognostic factor for either time to progression as metastatic disease (from detection of primary CRC to diagnosis of first distant metastasis; p = 0.009) or partial survival (from diagnosis of advanced disease to the time of death or last control; p = 0.006) or overall survival (p < 0.001). No significant impact on prognosis was observed for mutated *KRAS, NRAS,* and *PIK3CA* genes or combined *RAS* mutations (all *RAS*).

**Conclusions:**

Our study defines both prevalence and prognostic role of main activated oncogenes in a population-based large collection of CRC patients.

**Electronic supplementary material:**

The online version of this article (doi:10.1186/s12967-016-1053-z) contains supplementary material, which is available to authorized users.

## Background

Colorectal cancer (CRC) is the third most incident malignancy in both sexes, after lung and prostate cancer in men and breast and cervix cancer in women; it also represents the fourth most frequent neoplastic cause of death after lung, stomach, and liver cancer [1]. The disease is more common in men than women. In 2015, 132,700 new cases of colorectal cancer and 49,700 disease-related deaths were estimated in USA [2]. The percent of colon and rectum cancer deaths is highest among people aged 75-84 [2].

From the pathogenetic point of view, CRC is a complex and heterogeneous neoplastic disease, exhibiting multiple genetic and epigenetic alterations. The accumulation of acquired molecular aberrations is involved in colorectal tumorigenesis, able to transform normal colonic epithelieum into adenocarcinomas. Starting from the model proposed by Fearon and Vogelstein in 1990, not only tubular and tubulovillous adenoma may progress to invasive adenocarcinoma, but it is now recognized that the serrated adenomas (SSA) and traditional serrated adenomas (TSA), originally excluded, may also undergo malignant transformation [3, 4].

At least four kinds of different pathogenic mechanisms have been proposed to be relevant in CRC classification: chromosomal instability (CIN), microsatellite instability (MSI), CpG island methylator phenotype (CIMP) and global DNA hypomethylation. The CIN pathway is the most common feature, occurring in 85 % of colorectal cancers and showing numerical chromosome changes and/or multiple structural aberrations. Different studies suggest that CIN promotes cancer progression by increasing clonal diversity [5]. It involves mutations in the *APC* gene and/or the loss of chromosome 5q where it is located, mutations in the *KRAS* oncogene, the loss of chromosomal arm 18q, and deletion of chromosome 17p where is mapped the *TP53* tumour suppressor gene [6]. The MSI phenotype is present in the remaining 15 % of CRCs. It is usually mutually exclusive with CIN aberrations and involves the impairment of the DNA mismatch repair system. This damage causes replication errors at genomic level—detectable as numerical alterations in the repetitive units of DNA microsatellites—and inactivating mutations in target tumour suppressor genes [7]. The CIMP feature is characterized by hypermethylation of genomic DNA located in CpG islands, specific regulatory sites enriched in CpG motifs and mapped in the promoter regions of tumour suppressor genes. Although the mechanism is still under investigation, different studies suggest a pathogenetic correlation between CIMP and occurrence of BRAF mutations in CRC [7, 8]. Finally, a reduction of DNA methylation rates has also been reported in majority of CIN-positive CRCs [9].

Altogether, the above-mentioned genetic and epigenetic alterations have a powerful impact on different cellular functions: cell proliferation, apoptosis, differentiation, angiogenesis, invasion and immortalization [10, 11]. Among others, the EGFR-RAS-RAF-MEK-ERK and the PI3 K-AKT-mTOR signalling cascades play the main roles in CRC development and progression. In particular, activated RAS (mainly, KRAS and NRAS) proteins promote cell proliferation through constitutive stimulation of the downstream RAF-MEK-ERK effectors in the so-called mitogen-activated protein-kinase (MAPK) pathway [12].

In recent past years, the EGFR-depending pathway has been largely exploited for personalized therapies and, in particular, EGFR has become a key target of specific inhibitors to treat metastatic CRCs [13, 14]. Activating mutations in K-/N-RAS are recognized as a strong predictor of resistance to EGFR-targeted agents (monoclonal antibodies, mAbs), since they cause a constitutive phosphorylation of the RAS proteins—independent on activation status of the upstream EGFR protein—which in turn permanently promote cell proliferation and drastically reduce the effects of the EGFR inhibition [15–18].

The role of *RAS* mutations as prognostic factors in CRC patients remains uncertain and may be somehow dependent on populations’ origin [19–22]. Conversely, oncogenic BRAF mutations seem not to be predictive of insensitivity to the anti-EGFR therapy, but are recognized as predictors of poor survival [21, 23–27].

In Sardinia, whose population is genetically homogeneous due to its historical isolation and, thus, presents a high rate of inbreeding, colorectal cancer represents the second principal death-causing malignancy, with an incidence quite comparable with that observed in Western countries (standardized rate, 117.4 per 100.000 inhabitants per year; Sardinian population includes about one million and half inhabitants) [28, 29]. The contribution of *KRAS*, *BRAF,* and *PIK3CA* mutations to CRC pathogenesis was previously investigated by our group on a limited subset of patients from Sardinia [30]. The aim of this study was to evaluate the prognostic role of the somatic mutations in all candidate genes (*KRAS, BRAF, NRAS,* and *PIK3CA*) using a very large population-based collection of Sardinian patients with metastatic CRC.

## Methods

### Samples

One thousand two hundred and eighty-four patients with histologically-proven diagnosis of colorectal carcinoma (CRC) were included into the study. At the time of enrolment, only patients presenting with metastatic disease (stage IV, according to the American Joint Committee on Cancer (AJCC) guidelines [31]) entered the study. For all included patients, medical records and pathology reports were used to assess the histological classification and disease stage at the time of the diagnosis of the primary CRC. To avoid any bias, CRC patients were consecutively collected from September 2009 to December 2015; they were included regardless of age at diagnosis and disease characteristics of the primary tumour. No CRC case from our series was associated with clinically relevant colorectal polyposis. Sardinian origin was ascertained in all cases through verification of the place of birth for all patients and their parents.

Formalin-fixed, paraffin-embedded tissue samples from CRC patients were obtained from the archives of the Institutes and Services of Pathology participating to the study. Tissue sections were estimated to contain at least 80 % neoplastic cells by light microscopy.

All patients were informed about the aims of this study and, before the tissue sample was collected, gave a written informed consent. The study was reviewed and approved by the ethical review board of the University of Sassari.

### Mutation analysis

Genomic DNA was isolated from tissue sections using standard protocols, as previously described [28]. Briefly, somatic DNA was purified using the QIAamp DNA FFPE Tissue kit (Qiagen Inc., Valencia, CA, USA) and the DNA quality assessed for each specimen.

Mutation analysis was conducted in the coding sequence of the following genes: *KRAS* (exons 2, 3, and 4), *NRAS* (exons 2, 3, and 4), *BRAF* (exon 15, where almost all of the oncogenic mutations are located), and *PIK3CA* (exons 9 and 20, which are mostly implicated in the protein kinase activity). For *KRAS*, *NRAS*, and *BRAF* genes, quantitative measurements of mutations were based on pyrosequencing assays, which were performed on a PyroMark Q24 system (Qiagen Inc., USA), following the manufacturer’s instructions. For *PIK3CA* gene, exons 9 and 20 were investigated for mutations by direct sequencing, using an automated fluorescence-based cycle sequencer (ABIPRISM 3130, Life Technologies-ThermoFisher Scientific, Waltham, MA, USA), as previously described by our group [30]. Protocols for PCR-based assays will be available upon request. For *PIK3CA* gene, mutation analysis was missing in about two-fifths of cases (488/1284; 38 %) due to the low amount of available tumour tissue samples.

### Statistical analysis

The Cox regression model was performed using raw mortality and tumor-specific mortality. The time of overall survival was expressed in months, and the independent variables (*KRAS*, *NRAS*, all-RAS, *BRAF*, RAS-BRAF, *PIK3CA*, all genes, pT, pN, M) were stratified in age groups: 32–45 (N = 60), 46–64 (N = 440), and 65–98 (N = 491). Evaluation of the impact of all independent variables on prognosis was calculated regardless the type of treatment (EGFR inhibition or others) administered to patients. Kaplan–Meier estimates were executed through stratification by mutation data. The above-mentioned variables (mutational status, survivals, pT, pN, M) were included in a logistic regression for multivariate analysis by Pearson’s Chi Square test. The exact coefficient for sample proportion analysis was performed to determine whether there was any significant difference (below 0.05 level) between considering a mutated gene alone and considering mutations in multiple genes. All analyses were performed using the statistical package SPSS/7.5 per Windows.

## Results

Genomic DNA from primary tumour tissues of 1284 consecutively-collected patients with metastatic colorectal carcinoma (mCRC), originating from different geographical areas within Sardinia island, was screened for somatic mutations in *KRAS*, *NRAS*, *BRAF*, and *PIK3CA* genes. As reported in Table 1, the most frequent anatomical site and degree of differentiation of the primary tumour were the left colon (516; 40 %) and the moderately differentiated grade (1041; 81 %), respectively. The median age was 64 years (range, 32-88 years), with a preponderance of males (772; 60 %) (Table 1). At the time of diagnosis, minority of patients presented with localized disease (AJCC stage II: 286; 22 %) (Table 1).

**Table 1:** Distribution of RAS-BRAF mutations according to the characteristics of CRC patients

Characteristic	No.	%	*KRAS* mut	*NRAS* mut	*BRAF* mut	All mut
*Sex*						
Male	772	60.1	253 (32.8 %)	31 (4.0 %)	13 (1.7 %)	297 (39.8 %)
Female	512	39.9	204 (39.8 %)	22 (4.3 %)	14 (2.7 %)	240 (46.9 %)
*Anatomical tumour site*						
Right-transverse colon	441	34.3	148 (35.6 %)	18 (4.1 %)	9 (2.0 %)	175 (39.7 %)
Left colon	516	40.2	185 (35.9 %)	21 (4.1 %)	10 (1.9 %)	216 (41.9 %)
Rectum	327	25.5	124 (37.9 %)	14 (4.3 %)	8 (2.4 %)	146 (44.6 %)
*Disease stage at diagnosis*						
Stage II (T_3–4_N_0_M_0_)	286	22.3	108 (37.8 %)	11 (3.8 %)	6 (2.1 %)	125 (43.7 %)
Stage III (T_X_N_1–3_M_0_)	567	44.1	191 (33.7 %)	24 (4.2 %)	9 (1.6 %)	224 (39.5 %)
Stage IV (T_X_N_X_M_1_)	431	33.6	158 (36.7 %)	18 (4.2 %)	12 (2.8 %)	188 (43.6 %)
*Tumour grading*						
Well differentiated	138	10.7	49 (35.5 %)	7 (5.1 %)	3 (2.2 %)	59 (42.8 %)
Moderately differentiated	1041	81.1	370 (35.5 %)	41 (3.9 %)	21 (2.0 %)	432 (41.5 %)
Poorly differentiated	105	8.2	38 (36.2 %)	5 (4.8 %)	3 (2.9 %)	46 (43.8 %)
*Age, years*						
≤50	126	9.8	47 (37.3 %)	10 (7.9 %)	3 (2.4 %)	60 (47.6 %)
51–60	325	25.3	118 (36.3 %)	12 (3.7 %)	6 (1.8 %)	136 (41.8 %)
61–70	492	38.3	173 (35.2 %)	19 (3.9 %)	9 (1.8 %)	201 (40.9 %)
>70	341	26.6	119 (34.9 %)	12 (3.5 %)	9 (2.6 %)	140 (41.1 %)

**Fig. 1:**
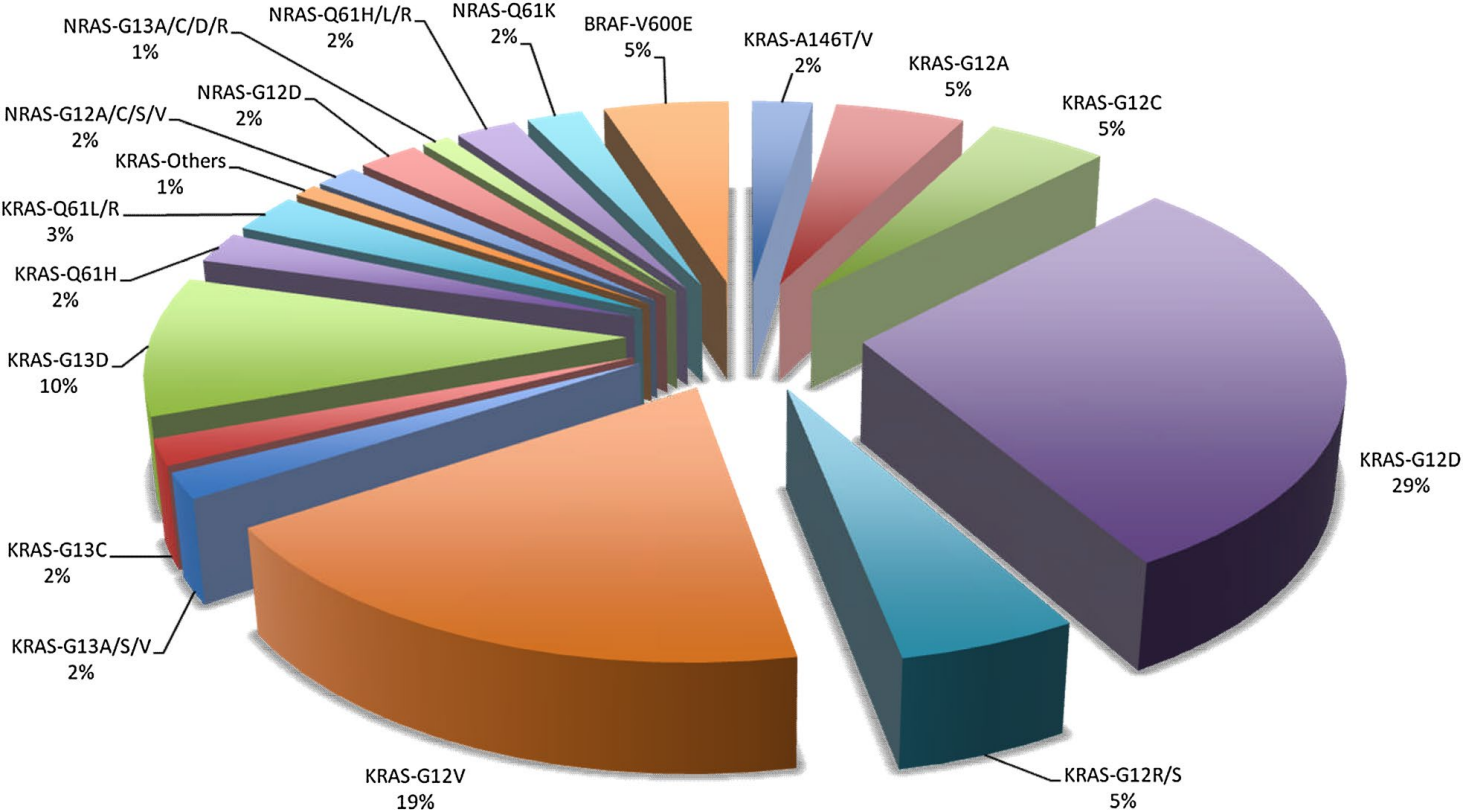
Somatic mutations in candidate genes among Sardinian CRC patients

Pyrosequencing assays were conducted for identification of mutations in the exons 2, 3 and 4 of both the *KRAS* and *NRAS* oncogenes as well as in the exon 15 of the *BRAF* oncogene. Somatic mutations in these genes were detected in 537 of 1284 (41.8 %) primary tumours: 457 (35.6 %) *KRAS*, 53 (4.1 %) *NRAS*, and 27 (2.1 %) *BRAF* mutations. Overall, no concurrent mutations of *KRAS*, *NRAS*, and *BRAF* genes were detected. Six patients had two mutations in *KRAS* gene: G12C and Q61H, G12D and Q61L, G12D and Q61H, G12 V and Q61H, G12 V and Q61R, G13 V and Q61H. In Fig. 1, spectrum and distribution of the 543 mutations identified is shown. The codon 12 of *KRAS* (345/463; 74.5 %) and the codon 61 of *NRAS* (25/53; 47 %) were the most affected in our series (Additional file 1: Table S1). For *BRAF*, all 27 patients carrying a mutation in this gene presented the substitution of valine by a glutamic acid at position 600 (V600E), which has been widely demonstrated to account for vast majority of the *BRAF* mutations reported in literature (Fig. 1; Additional file 1: Table S1) [32, 33].

All mutations identified in the present study are reported in both the Human Gene Mutation Database (HGMD) at http://www.hgmd.cf.ac.uk/ac/index.php and the catalogue of somatic mutations in cancer (COSMIC) at http://www.sanger.ac.uk/genetics/CGP/cosmic/.

Among available DNA samples, 796 primary tumours were also evaluated for the occurrence of pathogenetic mutations in exons 9 and 20 of the *PIK3CA* gene (see “Methods” section). Overall, *PIK3CA* mutations were detected in 114 (14.3 %) patients; one primary CRC tissue presented two mutations (E545G and Q1033L). Screening revealed the occurrence of mutations in three codons of exon 9 (95/115; 82.6 %) and four codons of exon 20 (20/115; 17.4 %) in the *PIK3CA* gene (Additional file 1: Table S2).

Again, all identified *PIK3CA* mutations have been reported in gene mutation databases (HGMD and COSMIC; see above) as commonly associated with CRC, with a recognized functional role of the corresponding mutated proteins.

Table 2 summarizes the distribution and relationship of the somatic mutations identified in the series of 796 CRC patients analysed in all four genes. Altogether, a mutation of at least one gene was discovered in about half (378/796; 47.5 %) of CRC cases; in other words, one out of two primary tumours displayed an extended wild-type genetic status, with lack of any pathogenic sequence variation in these four main candidate genes.

**Table 2:** Frequencies of gene mutations in the series of 796 patients screened for all four genes

Mutated genes	*KRAS*	*KRAS* + *PIK3CA*	*NRAS*	*NRAS* + *PIK3CA*	*BRAF*	*BRAF* + *PIK3CA*	*PIK3CA*	Wild-type
Cases	227	36	21	8	16	6	64	418
%	28.5	4.5	2.7	1.0	2.0	0.8	8.0	52.5

**Fig. 2:**
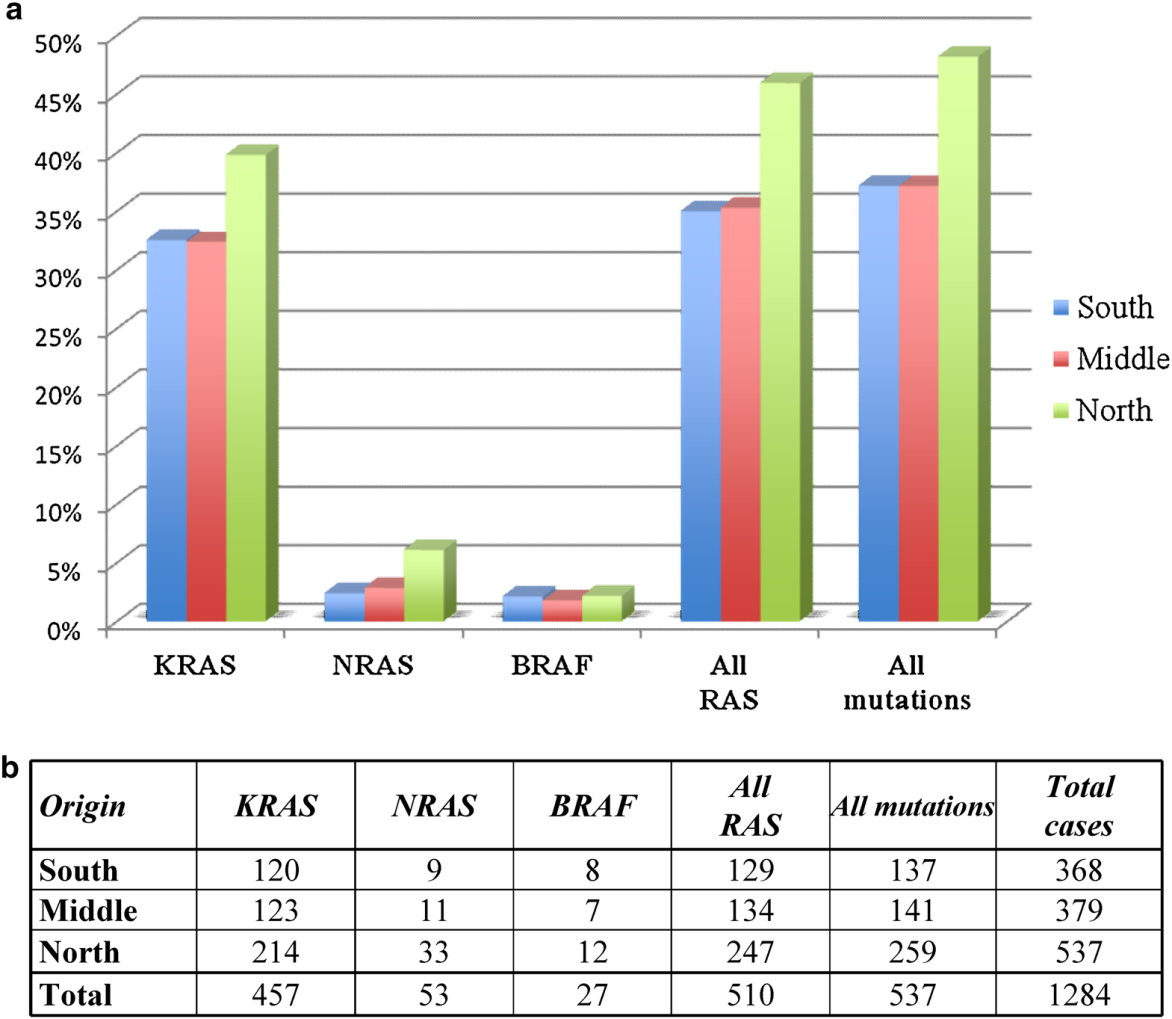
Geographical distribution of RAS and BRAF mutation carriers in Sardinia. **a** Frequencies in percentage; **b** number of cases

Considering the patients’ origin, distribution of *RAS* mutations was confirmed to be heterogeneous in Sardinia, as previously reported by our group [30]: 247/537 (46.0 %) mutated cases in North Sardinia versus 263/747 (35.2 %) in Middle-South Sardinia (Fig. 2). No difference in *BRAF* mutation distribution was instead observed within the island: 12/537 (2.2 %) in North Sardinia versus 15/747 (2.0 %) in Middle-South Sardinia (Fig. 2). It should be kept in mind, that all somatic samples from the entire series of Sardinian CRC patients were analysed for mutations with the same methodological procedures (see “Methods” section).

Mutations in *KRAS*, *NRAS*, *BRAF*, and *PIK3CA* genes were evaluated for association with several pathological parameters: sex, age at diagnosis, anatomical location of primary CRC, tumour grading, AJCC stage of the disease. No significant correlation was found between the occurrence of mutations in any of the four genes and all analysed parameters (see Table 1).

Logistic regression multivariate analysis was performed on the totality of tested patients to estimate relative risk of survival variations and to adjust potential confounding effects, as well as to assess possible multiplicative interactions. Using the Cox model adjusted according to disease stage and diagnosis age, the occurrence of mutations in *BRAF* gene was found to have a highly-significant negative impact on prognosis, on either partial survival (from the diagnosis of advanced disease to the time of death or last control, PS) [p = 0.006; HR: 3.21; 95 % CI: 1.38−7.44] or overall survival (from the disease onset to the time of death or last control, OS) [p < 0.001; HR: 4.12; 95 % CI: 2.49–6.81] (Table 3). No prognostic values on both survivals of either *KRAS* or *NRAS* or all *RAS* (*KRAS* + *NRAS*) or *PIK3CA* mutated status were instead observed in our series (Table 3).

**Table 3:** Statistical correlation of gene mutations with prognostic parameters

Mutated gene	Risk ratio	95 % CI	p
Partial survival (from metastatic disease onset to death or last control)			
*KRAS*	1.257	0.628–1.739	0.4906
*NRAS*	1.634	1.018–2.712	0.0763
All *RAS*	1.345	0.968–2.158	0.0924
*BRAF*	3.214	1.387–7.445	0.0064
All *RAS* + *BRAF*	1.733	0.997–2.994	0.0511
*PIK3CA*	1.403	0.877–1.479	0.1365
Overall survival (from disease diagnosis to death or last control)			
*KRAS*	1.219	0.983–1.512	0.0703
*NRAS*	1.052	0.612–1.810	0.8522
All *RAS*	1.108	0.932–1.316	0.2432
*BRAF*	4.120	2.491–6.812	<0.001
All *RAS* + *BRAF*	1.878	1.238–3.996	0.0394
*PIK3CA*	0.934	0.711–1.226	0.6246
Time to progression as metastatic disease (from disease diagnosis to first metastasis)			
*KRAS*	1.077	0.843–1.677	0.1508
*NRAS*	1.154	0.853–1.851	0.1006
All *RAS*	1.277	1.025–2.371	0.0943
*BRAF*	2.972	1.261–6.332	0.0091
All *RAS* + *BRAF*	2.217	1.125–4.371	0.0214
*PIK3CA*	0.964	0.691–1.346	0.8337

Considering the time to progression as metastatic disease (from the detection of primary CRC to the diagnosis of first distant metastasis, TTPM), the occurrence of *BRAF* mutations again remained a statistically-independent negative prognostic factor [p = 0.009; HR: 2.97; 95 % CI: 1.26–6.33] (Table 3). Noteworthy, the impact on OS and TTPM of the *BRAF* mutations was so highly significant to negatively affect such survivals in the combined group of mutated patients (considering all mutations in *RAS* and *BRAF* genes) (Table 3).

**Fig. 3:**
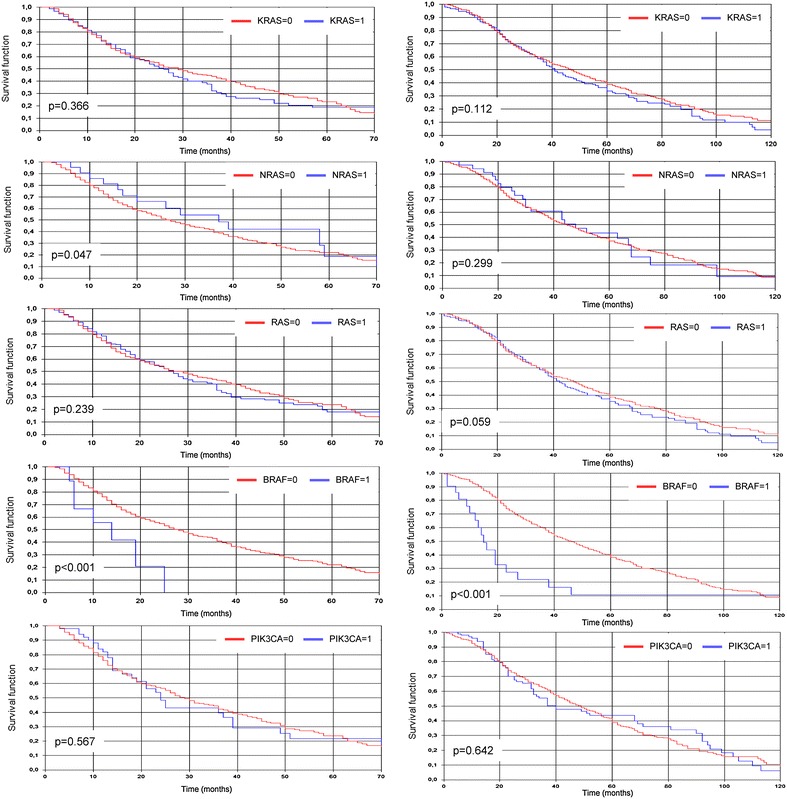
Kaplan-Meier cumulative survival analyses according to gene mutation status: partial survival in the *left* column, overall survival on the *right*; 0 wild-type, 1 mutated

Using the Kaplan–Meier method, survival curves indicated that patients carrying a *BRAF* mutation presented strongly significant poorer partial [p < 0.001] and overall [p < 0.001] survivals in comparison with those carrying a *BRAF* wild-type gene (Fig. 3). With the exception of the *NRAS* mutations on partial survival [p = 0.047], no significant association with both types of survival was instead observed for mutations in the other genes (a prognostic value close to be significant was found for all-*RAS* mutation-positive status [p = 0.059] on overall survival) (Fig. 3).

No correlation between occurrence of gene mutations and response to therapies (EGFR inhibitors or others) has been inferred into the present study (such clinical data are being collected for a successive study in next future).

## Discussion

Colorectal cancer is one of the most prevalent malignancies worldwide [1, 2, 28]. Great efforts have been made in the last decades for the comprehension of the pathophysiological and molecular mechanisms of the disease, and improvements in its clinical management have been made. Despite this, a lot more has to be done to improve the outcomes in the treatment of advanced stage patients, in whom the prognosis remains relatively poor [34]. On this purpose, the most relevant advances in recent years regard the targeted therapies inhibiting the EGFR-RAS cascade. The EGFR is a transmembrane protein for the epidermal growth (EGF) that explicates its functions through the activation of the RAS protein family (HRAS, KRAS, and NRAS). Activated RAS proteins promote cell proliferation and tumour progression and invasiveness through several mechanisms, including constitutive stimulation of the kinases into the MAPK pathway [15]. EGFR-targeted agents, like cetuximab and panitumumab that compete with EGF for binding to the receptor, have been employed in clinical practice, in order to reduce cell proliferation, migration, invasion, and metastasis formation [18].

*KRAS* mutations have been demonstrated to reduce the effectiveness of the anti-neoplastic agents mentioned, and they are currently a validated predictive marker of negative pharmacological response to anti-EGFR therapies. Mutations on codons 12 and 13 in exon 2 have been initially established as biomarkers of resistance to anti-EGFR antibodies; soon after, mutations on codons 61 and 146, as well as mutations on exons 3 and 4, have been discovered to produce the same effect [35–38]. As we mentioned before, these mutations cause a constitutive phosphorylation of the RAS proteins, which permanently promote cell proliferation and drastically reduce the effects of the EGFR inhibition [15–18]. Mutations in the entire coding region of the *NRAS* oncogene as well as in the exon 20 of the *PIK3CA* gene, which are rarer in CRC patients, have also been demonstrated to be predictors of reduced response to anti-EGFR therapies [36–38].

In our series, *KRAS*, *NRAS*, *BRAF*, and *PIK3CA* mutations were observed in 47.5 % of the CRC cases examined, mostly confirming the mutation prevalence observed in our previous study (a mutation of at least one among the *KRAS*, *BRAF*, and *PIK3CA* genes was discovered in about 45 % of Sardinian CRC cases) [30]. In Italian population, higher rates were recently reported in a considerably smaller collection of CRC patients from North Italy [39]. Using the same mutation screening tests of our present study (pyrosequencing assays; see “Methods” section), Foltran and colleagues reported that the 39.2 % of their cases was wild-type, defined as lack of mutations in *KRAS* exons 2-4, *NRAS* exons 2 and 3, *BRAF* exon 15, and *PIK3CA* exons 9 and 20 [39]. Conversely, the mutation rates were 47.4, 3.6, 5.2, and 16.5 % for *KRAS*, *NRAS*, *BRAF*, and *PIK3CA*, respectively (about 12 % of cases carried both *KRAS* and *PIK3CA* mutations) [39]. In the Sardinian population, mutation rates were much lower for *KRAS* (35.6 %) and BRAF (2.1 %), slightly lower for PIK3CA (14.3 %) and slightly higher for *NRAS* (4.1 %). Focusing on *KRAS* mutations only, Sardinian CRC patients were found to carry a mutated *KRAS* gene in 30 to 36 % of the cases [30, 40].

The distribution of the mutations within the territory of the island was confirmed to be heterogeneous, with the northern populations presenting higher rates of mutations, especially in the *RAS* genes [30]. This pattern confirms older evidences, regarding not only CRC but also other malignancies, and suggests that the genetic background may influence the occurrence of cancer gene mutations, even within relatively homogeneous populations such as that from Sardinia [30, 41–43]. Globally, the mutation rates found in our cohort are however within the ranges published in other series throughout the world [30, 44].

In our study, we did not evidence any relation between the mutation rates and the clinical and/or pathological parameters examined (age, sex, anatomical location, stage of the disease at diagnosis, grading). Most of the lesions were located in the left colon, but most of the mutations involved the rectum, even if the differences were not statistically significant. Some authors advocate that the anatomical location, histology and grading of the primary lesions has a relevant prognostic role, especially because *BRAF* mutations have been more frequently observed in right-sided, poorly differentiated mucinous tumors [26, 45–47].

Somatic mutations in the exon 15 of *BRAF* have been widely demonstrated, indeed, to negatively impact the prognosis of patients with CRC, independently of any clinical parameter studied [26, 45–47]. This was observed also in the present study; patients with substitution of a valine by a glutamic acid at position 600 (V600E), which was the only mutation found in our cases and the most commonly reported in the scientific literature, had a worst PS, OS, and TTPM in comparison to those without this alteration [32, 33]. Furthermore, the group of mutated patients—combining carriers of all mutually exclusive mutations in *RAS* and *BRAF* genes—presented a worst OS. This finding is in line with those of other authors who found that the all-wild type malignancies (*RAS*, *BRAF*, and in some cases *PIK3CA* included) had globally a better prognosis in comparison to the mutated ones [36, 39]. This is particularly relevant considering that a significant portion of the patients (approximately a half of them in our series) with CRC do not present any mutation of the genes mentioned above. The prognostic role of *PIK3CA* is less clear; it is known that it often coexists with *KRAS* mutations and predicts resistance to anti-EGFR therapies, but its exact prognostic role is poorly understood [39]. Ogino et al. evidenced that *PIK3CA* is associated with poor prognosis among patients with curatively resected colon cancer, but such an association was not confirmed neither in our series, nor in other studies in early or advanced stage patients [39, 48, 49].

The prognostic role of *KRAS* mutations as a global prognostic factor of disease progression and survival in CRC patients is controversial. Several studies have reported a statistically significant reduction in disease-free survival (DFS) and OS in the presence of *KRAS* mutations [50–52]. Ogino and colleagues performed an evaluation of the independent effect of the CIMP and MSI alterations as well as of the *KRAS* and *BRAF* mutations on the prognosis of 649 patients with IV stage CRC [53]. The study did not identify a relevant role for the *KRAS* mutations on prognosis, but the authors provided evidence that CIMP high appears to be an independent predictor of a low CRC specific mortality, while *BRAF* mutations are associated with a high CRC specific mortality [53]. By contrast, in their combined analysis of specific *KRAS* gene alterations, BRAF^V600E^ status, and MSI, Zlobec et al. found that specific *KRAS* mutations act as informative prognostic factors in both sporadic and hereditary CRC [54]. In another recent series of 342 cases, Winder et al. were the first to report an improved OS in patients with *KRAS* codon 13 mutations, compared to those with wild-type genes, further suggesting that different types of *KRAS* mutations may be differently associated with OS in patients with CRC [55]. Finally, a multivariate analysis by Bazan et al. in a series of 160 cases found that *KRAS* codon 13 mutations were associated with poorer OS but not DFS [56]. Finally, Ren et al. in a recent well-designed meta-analysis performed a careful selection of 23 articles published from 1992, including 25 sets of data with 4687 patients and 1364 (29 %) *KRAS*-mutated cases [57]. Among them, 9 papers indicated that *KRAS* mutations were associated with worse prognosis, 15 failed to demonstrate any statistically significant association between such mutations and prognosis, and, finally, one data set identified an improved survival rate for patents with CRC and *KRAS* mutations [57]. Globally, the authors found that *KRAS* mutations were not associated with CRC prognosis, either before or after adjustment for the effect of publication bias [57].

In a previous study on Sardinian patients, we observed an improved TTPM in male patients with CRC harbouring *KRAS* mutations; we hypothesized that such a finding may be associated with the specific types of *KRAS* mutations observed in males and females as consequence of exposure to different lifestyle factors [57]. Nevertheless, such a finding was not confirmed in the present study, performed in a considerably greater number of patients. On the other hand, we found in the present series that a mutated *NRAS* negatively affects partial survival, even if it did not show any impact on OS or TTPM. Global cases with *RAS* mutations had a prognostic value on PS and TTPM close to be significant, and this further enhances the above-mentioned observation that colorectal malignancies with wild-type status in all these candidate genes generally present a better prognosis.

The discrepancy of results among the mentioned studies on the prognostic role of *RAS* and *PIK3CA* mutations in CRC may be caused by some confounding factors. The most frequent are: (1) heterogeneity of the study populations; (2) discrepancies in study designs and methodologies; (3) strategies for mutational detection; (4) presence of underlying predisposing conditions (i.e. ulcerative colitis); (5) variability of the staging systems (Dukes, AJCC, different editions); and (6) differences in clinical management and treatment. The advantage of our study is to avoid several bias through a prospective collection of data on a consistent high number of patients from a homogeneous population, processed and analysed in a single institution. To date, we can consider as validated the prognostic role of *BRAF* mutations as well as that of the wild-type status in all main candidate genes. Further studies are probably necessary to better assess the prognostic role of the single *RAS* and *PIK3CA* mutations.

## Conclusions

Our findings evidence that approximately a half of the Sardinian patients with CRC present one or more mutations in one or more of the *KRAS*, *NRAS*, *BRAF*, and *PIK3CA* genes. The *KRAS* mutations are the most frequent, followed by those in *NRAS*, and *BRAF*, as observed in other studies, and they are strongly confirmed to be mutually exclusive. On the other hand, *PIK3CA* mutations often coexist with the *RAS* mutations. With the exclusion of mutations in any of the other genes, harbouring a mutated *BRAF* was demonstrated to negatively impact the prognosis—regardless the type of survival taken into consideration—in CRC patients from Sardinian population. This makes the role of the latter mutations clear; as for the others, further investigations are necessary to better comprehend their prognostic impact.

## Additional file

10.1186/s12967-016-1053-z Additional tables S1 and S2. the file contains two supplementary tables depicting the exact distribution of the 543 mutations identified in *RAS* and *BRAF* genes (Table S1), as well as the distribution of the 115 *PIK3CA* mutations (Table S2) in our cohort.

## Abbreviations

CIMP: CpG islant methylator phenotype
CIN: chromosomal instability
CRC: colorectal cancer
DFS: disease free survival
EGF: epidermal growth factor
EGFR: epidermal growth factor receptor
mAbs: monoclonal antibodies
MAPK: mitogen-activated protein-kinase
mCRC: metastatic colorectal cancer
MSI: microsatellite instability
OS: overall survival
PS: partial survival
SSA: serrated adenomas
TSA: traditional serrated adenomas
TTPM: time to metastatic progression
