# Supplementary material for: Prognostic impact of KRAS, NRAS, BRAF, and PIK3CA mutations in primary colorectal carcinomas: a population-based study
Source: J Transl Med. 2016 Oct 13;14:292. doi: 10.1186/s12967-016-1053-z (PMC5064898; doi:10.1186/s12967-016-1053-z)
Supplement: Supplementary file 1 — 10.1186/s12967-016-1053-z Additional tables S1 and S2. the file contains two supplementary tables depicting the exact distribution of the 543 mutations identified in RAS and BRAF genes (Table S1), as well as the distribution of the 115 PIK3CA mutations (Table S2) in our cohort. [file 12967_2016_1053_MOESM1_ESM.docx]

**Table S1.** Distribution of the 543 mutations identified in *RAS* and *BRAF* genes.

| ***Gene*** | ***Exon*** | ***Codon*** | ***Mutation protein*** | ***Mutation***  ***DNA*** | ***No. +ve cases*** | ***%*** |
| --- | --- | --- | --- | --- | --- | --- |
| *KRAS* | 2 | 12 | G12_G13insG | c.36_37insGGT | 1 | *0,2* |
|  |  |  | G12A | c.35G>C | 28 | *5,2* |
|  |  |  | G12C | c.34G>T | 26 | *4,8* |
|  |  |  | G12D | c.35G>A | 160 | *29,5* |
|  |  |  | G12R | c.34G>C | 7 | *1,3* |
|  |  |  | G12S | c.34G>A | 21 | *3,9* |
|  |  |  | G12V | c.35G>T | 102 | *18,8* |
|  |  | 13 | G13A | c.38G>C | 1 | *0,2* |
|  |  |  | G13C | c.37G>T | 10 | *1,8* |
|  |  |  | G13D | c.38G>A | 54 | *9,9* |
|  |  |  | G13S | c.37G>A | 7 | *1,3* |
|  |  |  | G13V | c.38G>T | 2 | *0,4* |
|  | 3 | 49 | E49K | c.145G>A | 1 | *0,2* |
|  |  | 59 | A59E | c.176C>A | 1 | *0,2* |
|  |  |  | A59T | c.175G>A | 1 | *0,2* |
|  |  | 61 | Q61H | c.183A>C | 12 | *2,2* |
|  |  |  | Q61L | c.182A>T | 8 | *1,5* |
|  |  |  | Q61R | c.182A>G | 7 | *1,3* |
|  | 4 | 117 | K117N | c.351A>T | 1 | *0,2* |
|  |  | 146 | A146T | c.436G>A | 11 | *2,0* |
|  |  |  | A146V | c.437C>T | 2 | *0,4* |
| *NRAS* | 2 | 12 | G12A | c.35G>C | 2 | *0,4* |
|  |  |  | G12C | c.34G>T | 2 | *0,4* |
|  |  |  | G12D | c.35G>A | 13 | *2,4* |
|  |  |  | G12S | c.34G>A | 3 | *0,6* |
|  |  |  | G12V | c.35G>T | 2 | *0,4* |
|  |  | 13 | G13A | c.38G>C | 1 | *0,2* |
|  |  |  | G13C | c.37G>T | 1 | *0,2* |
|  |  |  | G13D | c.38G>A | 1 | *0,2* |
|  |  |  | G13R | c.37G>C | 3 | *0,6* |
|  | 3 | 61 | Q61H | c.183A>C | 3 | *0,6* |
|  |  |  | Q61K | c.181C>A | 12 | *2,2* |
|  |  |  | Q61L | c.182A>T | 2 | *0,4* |
|  |  |  | Q61R | c.182A>G | 8 | *1,5* |
| *BRAF* | 15 | 600 | V600E | c.1799T>A (25)  c.1799_1800TG>AA (2) | 27 | *5,0* |

**Table S2.** Distribution of the 115 *PIK3CA* mutations

| ***Exon*** | ***Codon*** | ***Mutation protein*** | ***Mutation***  ***DNA*** | ***No. +ve cases*** | ***%*** |
| --- | --- | --- | --- | --- | --- |
| 9 | 542 | E542A | c.1625A>C | 2 | 1,7 |
|  |  | E542G | c.1625A>G | 3 | 2,6 |
|  |  | E542K | c.1624G>A | 9 | 7,8 |
|  | 545 | E545A | c.1634A>C | 56 | 48,7 |
|  |  | E545D | c.1635G>C | 1 | 0,9 |
|  |  | E545G | c.1634A>G | 7 | 6,1 |
|  |  | E545K | c.1633G>A | 12 | 10,4 |
|  | 546 | Q546E | c.1636C>G | 3 | 2,6 |
|  |  | Q546H | c.1638G>T | 1 | 0,9 |
|  |  | Q546K | c.1636C>A | 1 | 0,9 |
| 20 | 1035 | A1035V | c.3104C>T | 2 | 1,7 |
|  | 1043 | M1043I | c.3129G>A (3)  c.3129G>C (1) | 4 | 3,5 |
|  | 1045 | D1045N | c.3133G>A | 1 | 0,9 |
|  | 1047 | H1047R | c.3140A>G | 13 | 11,3 |
